# Draft genome sequence of *Enterococcus lactis* SP-50a, isolated from artisanal cheese, with potential to produce antimicrobial agents and biopolymers

**DOI:** 10.1128/mra.00434-26

**Published:** 2026-08-07

**Authors:** Itzel del Carmen Fonseca-Barrera, Carolina Peña-Montes, Karla Gabriela Álvarez-Villagomez, Silvia Portilla-Vázquez, Patricia Guillermina Mendoza-García

**Affiliations:** 1 Unidad de Investigación y Desarrollo en Alimentos (UNIDA), TecNM/Instituto Tecnológico de Veracruz, Veracruz, México; Nanchang University, Nanchang, Jiangxi, China

**Keywords:** *Enterococcus lactis*, genome, antimicrobial peptides, polyhydroxyalkanoate synthesis

## Abstract

Artisanal cheeses are a source of bacteria with biotechnological potential. The genome size of *Enterococcus lactis* SP-50a was 2.71 Mb with 2,637 genes. Analysis identified genes for bacteriocin and peptidoglycan hydrolase synthesis, associated with antimicrobial activity. Additionally, the *fabG* gene was detected, indicating a possible polyhydroxyalkanoate production.

## ANNOUNCEMENT

*Enterococcus lactis* could provide a dual biotechnological solution for reducing antimicrobial resistance (1) and plastic contamination (2). This genus synthesizes antimicrobial agents, including bacteriocins (3) and peptidoglycan hydrolases (4), and potentially harbors the metabolic routes for biopolymer biosynthesis (5, 6).

**Fig 1:**
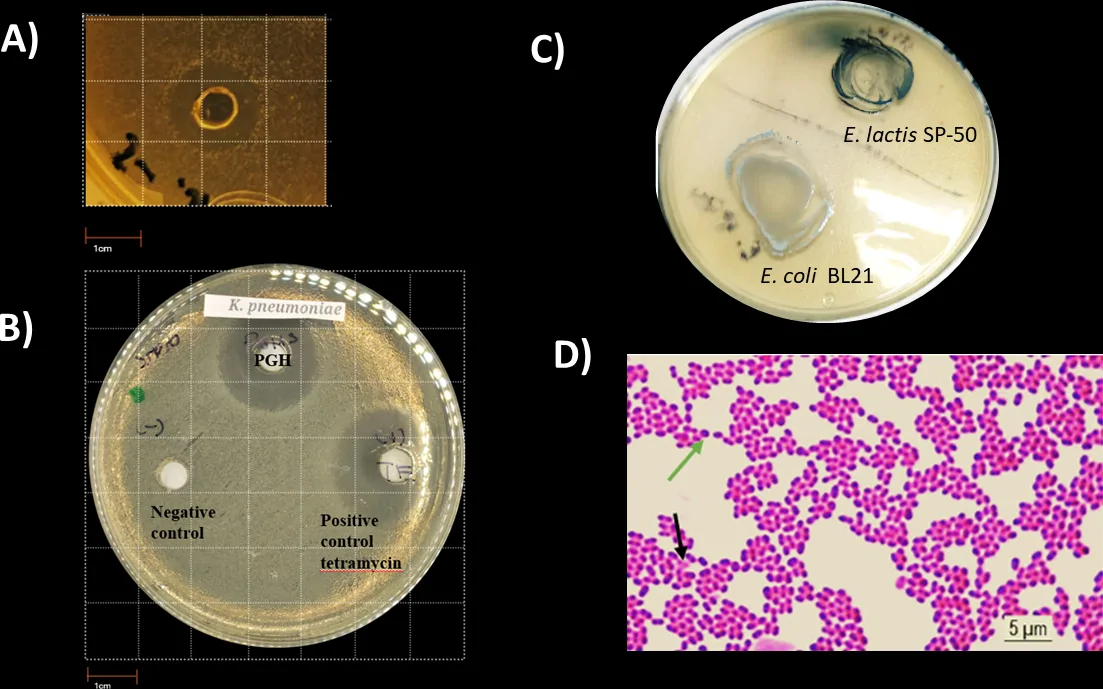
Preliminary testing of the antimicrobial activity assays, peptidoglycan hydrolase, and PHA production by the *Enterococcus lactis* SP-50a strain. (**A**) Inhibition zone of crude bacteriocin extract against *Listeria innocua* AST 062; (**B**) inhibition zone of peptidoglycan hydrolase against *Klebsiella pneumoniae* ATCC 10031; (**C**) colonies of *Enterococcus lactis* SP-50a and *E. coli* BL 21 stained with Sudan Black; (**D**) micrograph of *Enterococcus lactis* (SP-50a) stained with Sudan Black using an optical microscope at 100× (Leica Microsystems, Wetzlar, Germany): the green arrow points to a PHA-accumulating cell shown in blue-black, and the black arrow points to a non-PHA-accumulating cell showing the characteristic rose color.

The *E. lactis* SP-50a strain was isolated from cheese samples from local farms in the municipality of La Joya, Veracruz (19°36′36″ N, 97°01′32″ W), previously reported (7). The bacteria were grown in De Man, Rogosa and Sharpe (MRS) medium at 37°C for 12 h without agitation (OD_600nm_: 0.771 ± 0.02). The initial analysis of the bacteria showed 99.87% similarity to *Enterococcus faecium* DSM 20477 (ID: 99.87%, NR_114742). However, 16S sequencing is insufficient to distinguish between *E. faecium* and *E. lactis* (8). Whole-genome sequencing was performed for definitive species identification and for biotechnological applications. Genomic DNA was extracted using the Allprep Bacterial/DNA/RNA/Protein Kit (QIAGEN, catalog number 47054). DNA purity was confirmed via spectroscopy by BioTek Epoch 2 microplate spectrophotometer (A_260_/A_280_: 1.97; concentration: 219 ng/µL). DNA integrity was verified by 1% agarose gel electrophoresis to confirm the presence of high-molecular-weight genomic material. Library preparation was performed using the Rapid Sequencing Kit v14 (SQK-RAD114) following the manufacturer’s instructions. Briefly, 100–150 ng of genomic DNA was adjusted to a volume of 10 µL with nuclease-free water. Enzymatic fragmentation and adapter attachment were achieved through a transposase-based method using the fragmentation mix. The reaction was incubated at 30°C for 2 min, then heated to 80°C for 2 min to inactivate the enzyme and immediately cooled on ice. Finally, 1 µL of rapid adapter was added to the fragmented DNA and incubated at room temperature for 5 min. Basecalling was performed using Dorado V1.4 (https://github.com/nanoporetech/dorado) with the dna_r10.4.1_e8.2_400bps_sup@v4.3.0 model. Reads were filtered to retain those with a minimum Q score of 10 and a minimum length of 200 bp. The *de novo* genome was assembled using Flye v2.9.4 (9) and polished with Medaka v2.2.1 (https://github.com/nanoporetech/medaka). Both tools were run using default parameters unless otherwise specified. The assembly of *E. lactis* SP-50a resulted in a draft genome of 2,715,204 base pairs across two contigs: a 2.54 Mb bacterial chromosome (coverage: 153×) and a 172 kb plasmid (coverage: 151×). The genome was annotated using the NCBI Prokaryotic Genome Annotation Pipeline (PGAP) v.6.10. Genome assembly and annotation metrics are shown in Table 1. Taxonomic identity was confirmed using FastANI v1.34 tools (10), showing an average nucleotide identity of 99.26% against *E. lactis* strain SU-B46 (GCA_030490365.1). Several genes were identified as bacteriocin immunity protein (MHN6393358.1) and the class II bacteriocin (MHN6393359.1), involved in bacteriocin biosynthesis. Additionally, a lysozyme family protein (MHN6395638.1) and a glucosaminidase domain-containing protein (MHN6394094.1) have been identified, both of which belong to the peptidoglycan hydrolase family (11, 12). The *fabG* gene (MHN6393495.1), which can function as a *phaB* for polyhydroxybutyrate biosynthesis, was also identified (13). Preliminary analyses supporting the antimicrobial activity and PHB production of the *E. faecium* SP-50a strain are shown in Fig. 1.

**TABLE 1:** Assembly and genome annotation statistics for *Enterococcus lactis* SP-50a^*a*^

Parameters	Values
Assembly statistics	
Median read quality	22.5
Median read length (bp)	2611
Total base pairs (Mb)	418.96
Total reads	98,646
Number of contigs in assembly	2
N50 (Kb)	7.294
Genome metrics	
Genome size (bp)	2,715,204
G + C %	38
CDS (total)	2,547
CDSs (with protein)	2,417
CDSs (without protein)	130
Genes (total)	2,637
rRNA	6 (5s), 6 (16s), 6 (23s)
tRNA	68
Checkm completeness (%)	99.06
CheckM contamination (%)	0.94
SRA accession number	SRR37701720
GenBank accession number	JBWDIX000000000.1
BioProject	PRJNA224116
BioSample	SAMN56532058

^*a*^ The assembly statistics were obtained using NanoPlot v 1.46.2 (14) and annotated using the NCBI Prokaryotic Genome Annotation Pipeline (PGAP) V. 6.10.

## Data Availability

The Whole Genome Shotgun project has been deposited at DDBJ/ENA/GenBank under the accession number JBWDIX000000000.1. The raw sequencing data generated in this study have been deposited in the NCBI Sequence Read Archive database under accession number SRR37701720.

## References

[1] Chiș AA, Rus LL, Morgovan C, Arseniu AM, Frum A, Vonica-Țincu AL, Gligor FG, Mureșan ML, Dobrea CM. 2022. Microbial resistance to antibiotics and effective antibiotherapy. Biomedicines 10:1121. doi:10.3390/biomedicines10051121 35625857 PMC9138529

[2] Sheriff SS, Yusuf AA, Akiyode OO, Hallie EF, Odoma S, Yambasu RA, Thompson-Williams K, Asumana C, Gono SZ, Kamara MA. 2025. A comprehensive review on exposure to toxins and health risks from plastic waste: challenges, mitigation measures, and policy interventions. Waste Manag Bull 3:100204. doi:10.1016/j.wmb.2025.100204

[3] Wu Y, Pang X, Wu Y, Liu X, Zhang X. 2022. Enterocins: classification, synthesis, antibacterial mechanisms and food applications. Molecules 27:2258. doi:10.3390/molecules27072258 35408657 PMC9000605

[4] Shockman GD. 1992. The autolytic ('suicidase’) system of *Enterococcus hirae*: from lysine depletion autolysis to biochemical and molecular studies of the two muramidases of *Enterococcus hirae* ATCC 9790. FEMS Microbiol Lett 100:261–267. doi:10.1111/j.1574-6968.1992.tb14050.x 1362171

[5] Bhuwal AK, Singh G, Aggarwal NK, Goyal V, Yadav A. 2013. Isolation and screening of polyhydroxyalkanoates producing bacteria from pulp, paper, and cardboard industry wastes. Int J Biomater 2013:752821. doi:10.1155/2013/752821 24288534 PMC3830821

[6] Sundaram M, Radhakrishnan L, Chithiraivelu S, Athiyappa Gounder P. 2016. Enhanced production of polyhydroxybutyrate using *Enterococcus faecium* KT722772 isolated from cattle rumen fluid by substrates optimization . Polym Plast Technol Eng 55:1908–1915. doi:10.1080/03602559.2016.1185616

[7] Portilla-Vázquez S, Rodríguez A, Ramírez-Lepe M, Mendoza-García PG, Martínez B. 2016. Biodiversity of bacteriocin-producing lactic acid bacteria from mexican regional cheeses and their contribution to milk fermentation. Food Biotechnol 30:155–172. doi:10.1080/08905436.2016.1198263

[8] Belloso Daza MV, Almeida-Santos AC, Novais C, Read A, Alves V, Cocconcelli PS, Freitas AR, Peixe L. 2022. Distinction between *Enterococcus faecium* and *Enterococcus lactis* by a *gluP* PCR-based assay for accurate identification and diagnostics. Microbiol Spectr 10:e03268-22. doi:10.1128/spectrum.03268-22 36453910 PMC9769498

[9] Kolmogorov M, Yuan J, Lin Y, Pevzner PA. 2019. Assembly of long, error-prone reads using repeat graphs. Nat Biotechnol 37:540–546. doi:10.1038/s41587-019-0072-8 30936562

[10] Jain C, Rodriguez-R LM, Phillippy AM, Konstantinidis KT, Aluru S. 2018. High throughput ANI analysis of 90K prokaryotic genomes reveals clear species boundaries. Nat Commun 9:5114. doi:10.1038/s41467-018-07641-9 30504855 PMC6269478

[11] Wohlkönig A, Huet J, Looze Y, Wintjens R. 2010. Structural relationships in the lysozyme superfamily: significant evidence for glycoside hydrolase signature motifs. PLoS One 5:e15388. doi:10.1371/journal.pone.0015388 21085702 PMC2976769

[12] Vermassen A, Leroy S, Talon R, Provot C, Popowska M, Desvaux M. 2019. Cell wall hydrolases in bacteria: insight on the diversity of cell wall amidases, glycosidases and peptidases toward peptidoglycan. Front Microbiol 10:331. doi:10.3389/fmicb.2019.00331 30873139 PMC6403190

[13] Zhang H, Liu Y, Yao C, Cao X, Tian J, Xue S. 2017. FabG can function as PhaB for poly-3-hydroxybutyrate biosynthesis in photosynthetic cyanobacteria *Synechocystis* sp. PCC 6803. Bioengineered 8:707–715. doi:10.1080/21655979.2017.1317574 28494182 PMC5736341

[14] De Coster W, D’Hert S, Schultz DT, Cruts M, Van Broeckhoven C. 2018. NanoPack: visualizing and processing long-read sequencing data. Bioinformatics 34:2666–2669. doi:10.1093/bioinformatics/bty149 29547981 PMC6061794

